# Machine learning based immune evasion signature for predicting the prognosis and immunotherapy benefit in stomach adenocarcinoma

**DOI:** 10.3389/fcell.2025.1656367

**Published:** 2025-09-25

**Authors:** Wenwu Xue, Guanglin Zhang, Cui Yang, Tingting Tan, Weichun Zhang, Hongcai Chen

**Affiliations:** ^1^ Department of Internal Medicine, Cancer Hospital of Shantou University Medical College, Shantou, China; ^2^ Department of Gynaecology and Obstetrics, Shantou Central Hospital, Shantou, China; ^3^ Department of Internal Medicine, Jinping District People’s Hospital of Shantou, Shantou, China

**Keywords:** stomach adenocarcinoma, immune evasion, machine learning, immunotherapy, prognostic signature

## Abstract

**Background:**

Stomach adenocarcinoma (STAD) remains a major contributor to cancer-related mortality worldwide. Despite advances in immunotherapy, only a subset of STAD patients benefits from immune checkpoint inhibitors, largely due to tumor-intrinsic immune evasion mechanisms. Therefore, robust predictive biomarkers are urgently needed to guide prognosis assessment and therapeutic decision-making.

**Methods:**

An integrative machine learning framework incorporating 10 algorithms was applied to construct an immune evasion signature (IES) using 101 model combinations. The optimal model was selected based on concordance index (C-index) across validation datasets. The prognostic and immunological relevance of the IES was assessed via survival analyses, immune infiltration deconvolution, and multiple immunotherapy response metrics. Key genes were further validated using qPCR, immunohistochemistry, and *in vitro* functional assays.

**Results:**

A four-gene IES developed via the LASSO method demonstrated robust prognostic power across TCGA and multiple external cohorts. High IES score were associated with poor survival, reduced immune cell infiltration (e.g., CD8^+^ T cells, dendritic cells), elevated M2 macrophage abundance, and an immunosuppressive tumor microenvironment. Patients in the low IES score group exhibited favorable immunotherapy-associated features, including higher TMB, lower TIDE scores, and increased response rates in three independent immunotherapy datasets. Additionally, the IES stratified patients by sensitivity to chemotherapy and targeted therapies. KLF16, one of the signature genes, was upregulated in STAD and promoted cancer cell proliferation *in vitro*.

**Conclusion:**

We established a novel IES with strong potential to predict prognosis and immunotherapy response in STAD. This IES may serve as a valuable tool for risk stratification and individualized treatment planning in clinical practice.

## 1 Introduction

Stomach adenocarcinoma (STAD) is one of the most prevalent malignancies worldwide and remains a leading cause of cancer-related mortality (Luo et al., 2022). There are around 1,089,103 new STAD cases reported and an estimated 768,793 deaths in 2020 globally (Sung et al., 2021). Despite significant advances in surgery, chemotherapy, targeted therapy, and immunotherapy, the prognosis for advanced STAD patients remains unsatisfactory due to tumor heterogeneity and resistance to treatment (Yasuda and Wang, 2024). Recent clinical applications of immune checkpoint inhibitors have offered new hope, but only a subset of patients derives substantial benefit, largely due to tumor-intrinsic mechanisms of immune evasion (Guan et al., 2023).

Immune evasion refers to the ability of tumor cells to escape immune surveillance, often through downregulation of antigen presentation, overexpression of immune checkpoint molecules, or alterations in the tumor microenvironment that inhibit effective immune responses. In STAD, the immunosuppressive microenvironment—characterized by regulatory T cells, M2 macrophages, and myeloid-derived suppressor cells—plays a pivotal role in immune escape and therapeutic resistance (Wang et al., 2024; Li et al., 2025). This immunosuppressive environment facilitates tumor immune escape, allowing cancer cells to evade host immune clearance. Thus, investigating genes associated with immune escape within the STAD immune microenvironment and developing corresponding models to forecast immunotherapy effectiveness hold significant importance for advancing immunotherapeutic strategies.

Given the complex interplay between immune evasion and immunotherapy responsiveness, there is an urgent need to develop robust molecular signatures that can capture immune escape traits and accurately predict patient prognosis and immunotherapy benefits. With the advent of high-throughput sequencing and machine learning techniques, it is now possible to integrate multi-omics data and build predictive models with high clinical utility.

In this study, we applied an integrative machine learning framework to develop a novel immune evasion-related signature (IES) in STAD, aiming to stratify patient risk, evaluate the tumor immune landscape, and predict responses to immunotherapy. This immune evasion signature may serve as a valuable tool for personalized prognosis assessment and therapeutic decision-making in STAD.

## 2 Materials and methods

### 2.1 Data collection and preprocessing

Transcriptomic profiles and corresponding clinical information for STAD patients (n = 325) were downloaded from The Cancer Genome Atlas (TCGA) database. Additional validation cohorts were retrieved from the Gene Expression Omnibus (GEO), including GSE84437 (n = 423), GSE62254 (n = 297), GSE15459 (n = 175), and GSE26253 (n = 432). ComBat algorithm was used for batch effect correction using the “sva” package, followed by probe-to-gene mapping and z-score scaling to ensure comparability across datasets (Leek et al., 2012). In brief, we included histologically confirmed STAD primary tumors with available baseline transcriptomic data and overall survival (OS) information. We excluded samples lacking survival time or status, duplicate entries, and cases with OS follow-up <90 days (deaths and censorings, 8 cases excluded in the TCGA STAD cohort, 12 cases excluded in the GSE84437 cohort, 6 cases excluded in the GSE84437 cohort, 5 cases excluded in the GSE84437 cohort, and 8 cases excluded in the GSE84437 cohort) (Feng et al., 2023), to ensure a minimal, clinically meaningful follow-up window. Additionally, three immunotherapy datasets—GSE91061 (n = 98, melanoma), GSE78220 (n = 28, melanoma), and the IMvigor210 cohort (n = 298, urothelial cancer)—were utilized to evaluate the predictive value of the IES in determining the effectiveness of immunotherapy. Differentially expressed genes (DEGs) between STAD and normal tissues were identified using the “limma” package, with a threshold of |Log2FC| > 1.5 and p-value <0.05. A curated list of immune evasion-related genes (IRGs) was obtained from prior studies and public databases (Lawson et al., 2020; Wen et al., 2025), which are presented in Supplementary Table S1.

### 2.2 Integrative machine learning algorithms constructed an optimal IES

To screen for prognostic IRGs, univariate Cox proportional hazards regression was applied to the TCGA-STAD cohort. Genes significantly associated with overall survival (*p* < 0.05) were retained as candidate prognostic biomarkers for subsequent model construction. We then subjected the identified biomarkers to an integrative machine learning framework to develop a robust prognostic IES. This framework incorporated 10 machine learning techniques, including random survival forests, elastic net, Lasso, Ridge regression, stepwise Cox regression, CoxBoost, partial least squares regression for Cox models, supervised principal component analysis, generalized boosted regression modeling, and survival support vector machines. Following the framework outlined in previous studies (Liu et al., 2022; Li Z. et al., 2023), 101 algorithmic combinations were evaluated in the TCGA cohort via leave-one-out cross-validation. Each model was subsequently validated in GEO cohorts, and the Harrell’s concordance index (C-index) was calculated across all datasets. The model with the highest average C-index was selected as the optimal IES.

### 2.3 Evaluation of the performance of IES

The optimal cut-off value for the IES score was determined using the “surv_cutpoint” function from the R package “survminer”. Patients were classified into high- and low-risk groups. Kaplan–Meier survival analysis and time-dependent ROC curves (via the “survivalROC” R package) were used to assess prognostic performance. We conducted both univariate and multivariate Cox regression analyses to identify prognostic factors in STAD patients. Based on these results, a prognostic nomogram was constructed using the “nomogramEx” R package, incorporating the IES-derived risk score and additional clinical variables. The predictive performance of the nomogram was assessed by comparing the predicted survival probabilities with the actual outcomes, which was illustrated using calibration plots.

### 2.4 Immune microenvironment and immunotherapy evaluation

To evaluate the immune microenvironment in STAD, we first applied the ESTIMATE algorithm to compute immune and stromal scores for each patient (Yoshihara et al., 2013). Additionally, seven other deconvolution tools—namely TIMER, xCell, MCP-counter, CIBERSORT, CIBERSORT-ABS, EPIC, and quanTIseq—were employed to estimate the composition of immune cells within tumor samples, selected for their complementary strengths (Li et al., 2020). The expression patterns of human leukocyte antigen (HLA)-associated genes and immune checkpoint regulators were illustrated using the “ggpubr” and “ggplot2” R packages. Furthermore, we conducted single-sample gene set enrichment analysis (ssGSEA) via the “GSVA” package to quantify immune-related functions and the infiltration levels of specific immune cell types.

### 2.5 Drug sensitivity evaluation

To explore the predictive capability of the IES model in assessing immunotherapy response, we analyzed several key metrics: tumor immune dysfunction and exclusion (TIDE) (Fu et al., 2020), immunophenoscore (IPS) (Charoentong et al., 2017), and tumor mutation burden (TMB) (Samstein et al., 2019). TMB score of TCGA STAD patients were calculated with “maftools” package. Elevated TIDE and immune escape scores, alongside reduced IPS and TMB values, were considered indicative of stronger immune evasion and diminished likelihood of response to immune checkpoint blockade. Drug response for each STAD case was predicted based on half-maximal inhibitory concentration (IC50) values calculated using the “oncoPredict” R package (Maeser et al., 2021), referencing data from the Genomics of Drug Sensitivity in Cancer (GDSC) database (Yang et al., 2013). Lower IC50 values represent increased sensitivity to therapeutic agents.

### 2.6 Protein expression data from the human protein atlas

Protein expression levels of genes included in the IES were extracted from the Human Protein Atlas (https://www.proteinatlas.org/) (Colwill et al., 2011), which integrates proteomic and transcriptomic data to chart protein localization across human tissues and tumor types. Expression profiles were obtained from both normal tissue and cancer-specific datasets.

### 2.7 Cell lines and gene silencing of KLF16

Normal gastric epithelial (RGM-1) and STAD cell lines (NCI-N87, CRL-5822, RPMI1640, BGC-823, and HGC-27) were acquired from the Shanghai Institute of Biochemistry and Cell Biology. All cells were maintained in ATCC-recommended media supplemented with fetal bovine serum (FBS; Gibco) and 1% penicillin-streptomycin (Sigma-Aldrich), and cultured at 37 °C in a humidified atmosphere containing 5% CO_2_. For gene silencing experiments, NCI-N87 and HGC-27 cells were transfected with KLF16-targeted siRNA or negative control siRNA using Lipofectamine 3000 (Invitrogen) according to the manufacturer’s instructions.

### 2.8 Quantitative PCR and cell proliferation assays

Total RNA was isolated using TRIzol reagent (Takara Bio), followed by cDNA synthesis using oligo(dT) primers. Quantitative real-time PCR (RT-qPCR) was performed on an ABI 7900HT system (Thermo Fisher Scientific) with SYBR Premix Ex Taq (Takara Bio), and gene expression levels were normalized to GAPDH.

For proliferation assessment, gastric cancer cells were seeded into 96-well plates at a density of 5,000 cells per well. At indicated time points, Cell Counting Kit-8 (CCK-8; Beyotime) reagent was added, and optical density (OD) values were measured. The proliferation index was calculated as the OD ratio at each time point relative to baseline.

## 3 Results

### 3.1 Identification of prognostic immune evasion-related genes in STAD

A total of 2962 DEGs between STAD and normal tissues were identified (Supplementary Figure S1A). By overlapping these DEGs with previously defined IRGs, we obtained a subset of 40 candidate genes (Supplementary Figure S1B). Univariate Cox regression analysis further revealed 8 IRGs (KLF16, ANXA5, TAP1, DOT1L, PRKCSH, NRP1, MARCKS, and AKR1B1) significantly associated with overall survival in STAD, which were considered as potential prognostic biomarkers (Supplementary Figure S1C).

### 3.2 Development of the IES using machine learning

**FIGURE 1:**
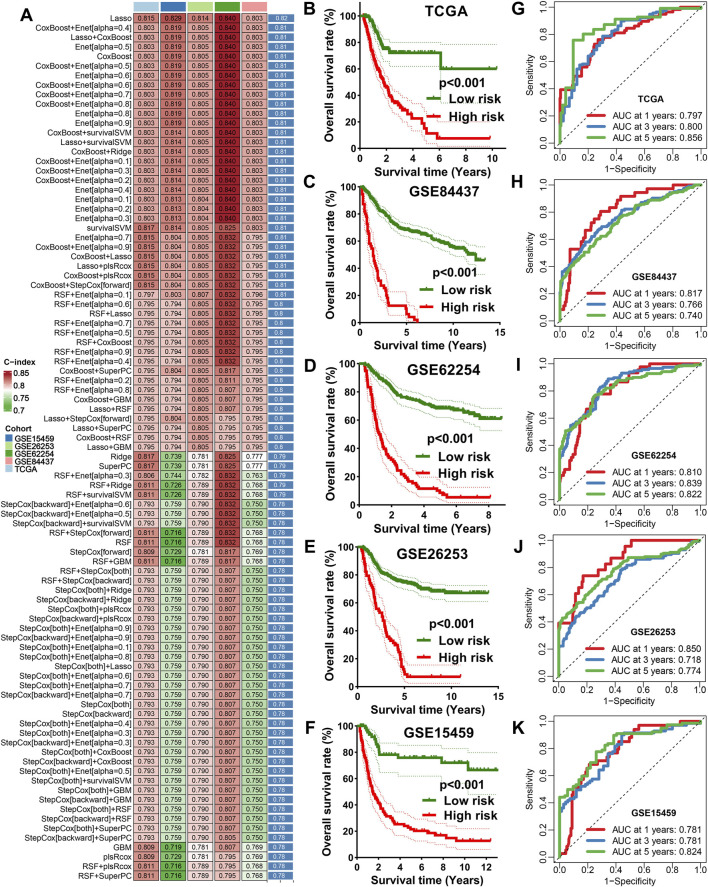
Integrative machine learning algorithms based IES. **(A)** The C-index of 101 kinds prognostic models developed by 10 machine learning algorithms in TCGA and GEO datasets. The survival curve of STAD patients with different risk score **(B–F)** and their corresponding ROC curve in TCGA, GSE84437, GSE62254, GSE15459, and GSE26253 cohort **(G–K)**.

To construct a robust prognostic model, we applied ten different machine learning algorithms and developed 101 predictive models across TCGA and GEO cohorts. The model generated by the LASSO algorithm achieved the highest average C-index (C-index = 0.82) and was selected as the optimal IES (Figure 1A). In LASSO algorithm, four genes (KLF16, ANXA5, TAP1, and DOT1L) were selected from above 8 potential prognostic biomarkers for the construction of optimal IES. The IES score (risk score) was calculated with the following formula: IES score = (0.282) × KLF16^exp^ + (−0.098) × ANXA5^exp^ + (−0.125) × TAP1^exp^ + (−0.035) × DOT1L^exp^. Based on the optimal cut-off value (0.035), STAD patients were divided into high- and low-risk groups. Kaplan–Meier survival analysis demonstrated significantly poorer overall survival in the high-risk group across all validation cohorts (TCGA, GSE84437, GSE62254, GSE15459, and GSE26253) (Figures 1B–F). The corresponding ROC curves indicated that the IES exhibited excellent predictive accuracy for 1-, 3-, and 5-year survival (Figures 1G–K).

### 3.3 Predictive power of IES for clinical outcomes

**FIGURE 2:**
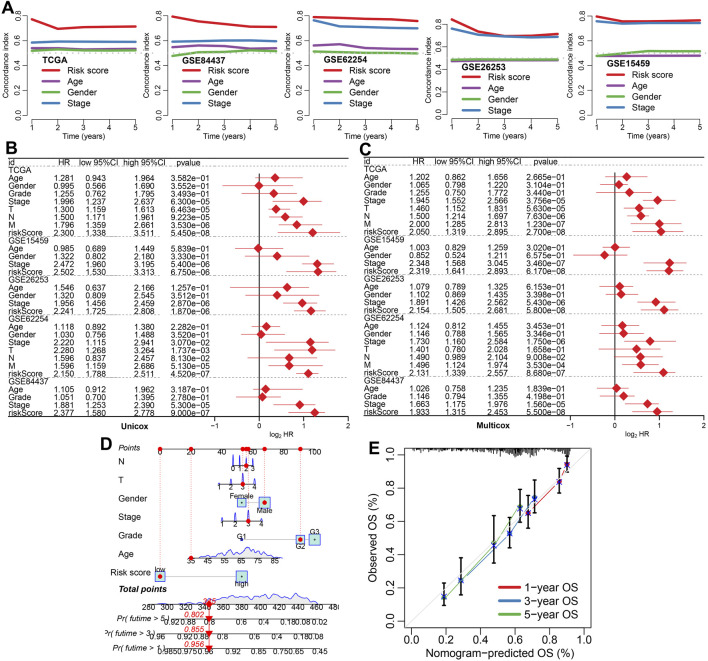
The performance of IES in predicting clinical outcome of STAD patients. **(A)** The C-index values for the IES, age, gender, and clinical stage for predicting the prognosis of STAD patients in TCGA and GEO datasets. **(B,C)** Risk factors for the prognosis of STAD patients identified by univariate and multivariate Cox regression analyses. **(D,E)** A predictive nomogram was developed and calibrated to assess the overall survival rates of STAD patients, providing a comprehensive evaluation of prognostic accuracy.

We compared the prognostic performance of the IES with traditional clinical factors such as age, gender, and stage. The IES consistently demonstrated a higher C-index across all datasets (Figure 2A). Both univariate and multivariate Cox regression analyses confirmed the IES as an independent risk factor for STAD prognosis (Figures 2B,C). A prognostic nomogram incorporating the IES and clinical parameters was constructed (Figure 2D), and calibration plots revealed excellent concordance between predicted and observed survival outcomes (Figure 2E).

### 3.4 Immune landscape associated with IES

**FIGURE 3:**
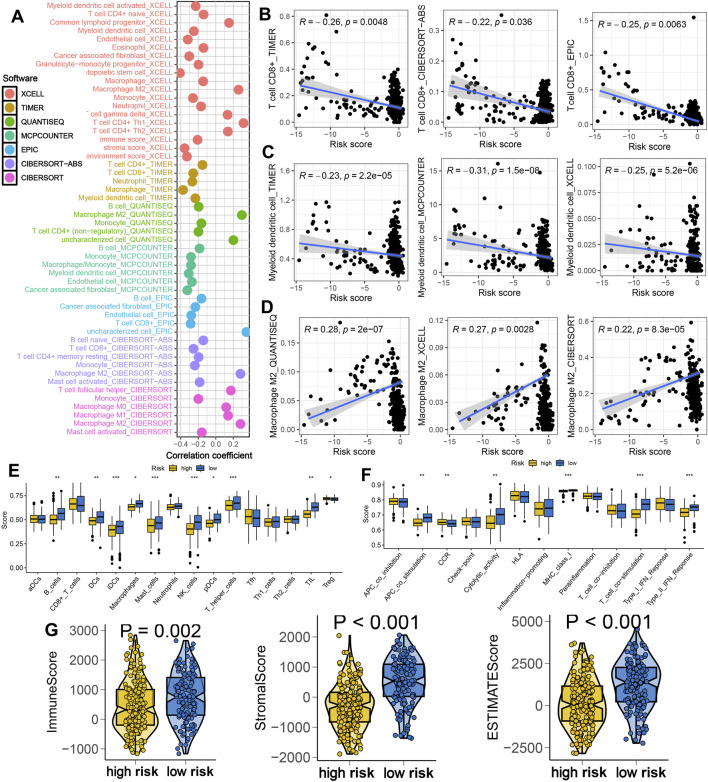
IES-based immune infiltration landscape in STAD. **(A)** Correlation between IES-based risk score and immune infiltration in STAD in the result of seven advanced algorithms. **(B–D)** Correlation between risk score and the abundance of CD8^+^ T cells, dendritic cells and macrophage M2. **(E,F)** Difference in the levels of immune cells and immune-related functions in different IES-based risk score groups. **(G)** Difference in the levels of immune score, stroma score, and ESTIMATE score in different IES-based risk score groups. *p < 0.05, **p < 0.01, ***p < 0.001.

To investigate the immunological implications of IES, we assessed the immune cell infiltration profiles using seven deconvolution algorithms. The IES-based risk score was negatively correlated with immune infiltration, including CD8^+^ T cells and dendritic cells, while positively correlated with immunosuppressive M2 macrophages (Figures 3A–D). Patients in the low-risk group exhibited higher enrichment scores for immune cells and immune-related functions, such as iDCs, mast cells, NK cells, TILs, cytolytic activity and T cell co-stimulation (Figures 3E,F). Moreover, they had significantly higher immune, stromal, and ESTIMATE scores, indicating a more active immune microenvironment (Figure 3G).

### 3.5 Association between IES and immunotherapy response

**FIGURE 4:**
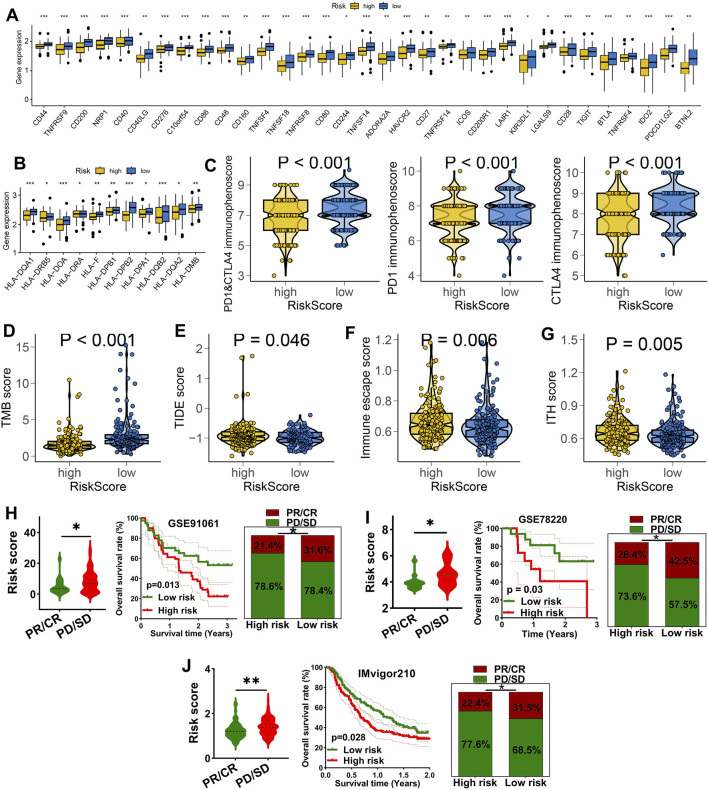
IES as a biomarker for predicting the immunotherapy response in STAD. **(A,B)** Difference in the level of immune checkpoints **(A)**, HLA-related genes **(B)**, PD1&CTLA4 immunophenoscore **(C)**, TMB score **(D)**, TIDE score **(E)**, immune escape score **(F)**, and ITH score **(G)** in different IES-based risk score groups. The immunotherapy response and overall rate in patients with high and low ORS score in GSE91061 **(H)**, GSE78220 **(I)** and IMvigor210 **(J)** datasets. *p < 0.05, **p < 0.01, ***p < 0.001.

We further explored the role of IES in predicting immunotherapy benefits. Patients in the low IES group showed increased expression of immune checkpoint molecules and HLA genes (Figures 4A,B), elevated PD1 & CTLA4 immunophenoscore (Figure 4C), and higher TMB scores (Figure 4D). Elevated expression of HLA-related genes and TMB score predicted a higher chance of immunotherapy benefits (Lin and Yan, 2021; Hodi et al., 2021). A stronger response to immunotherapy was indicated by low TIDE and immune escape scores (Fu et al., 2020; Lin et al., 2020). Conversely, these patients had significantly lower TIDE scores, immune escape scores, and intra-tumor heterogeneity (ITH) scores (Figures 4E–G), suggesting a reduced potential for immune evasion. In three independent immunotherapy cohorts (GSE91061, GSE78220, IMvigor210), lower IES scores were consistently associated with better response rates and improved survival outcomes (Figures 4H–J).

### 3.6 Drug sensitivity analysis based on IES

**FIGURE 5:**
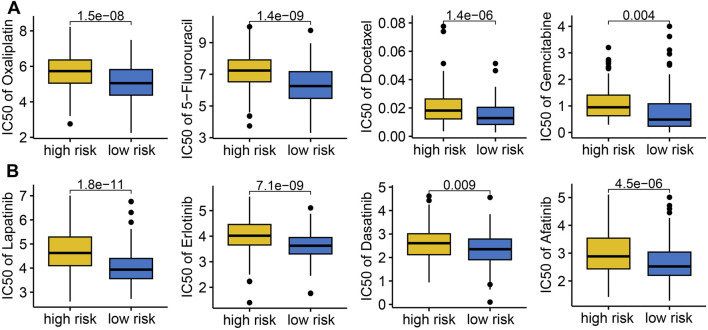
The IC50 value of common drugs in different IES-based risk score groups. Low IES score indicated higher IC50 values for commonly used drugs in both chemotherapy **(A)** and targeted therapy **(B)**.

We evaluated the association between IES and drug sensitivity. Patients with low IES-based risk score exhibited significantly lower IC50 values for multiple chemotherapeutic agents (e.g., Cisplatin, Oxaliplatin, Docetaxel, Gemcitabine) and targeted therapies (e.g., Lapatinib, Erlotinib, Dasatinib, Afatinib), suggesting greater sensitivity to conventional treatments (Figures 5A,B).

### 3.7 IES and cancer-related biological pathways

**FIGURE 6:**
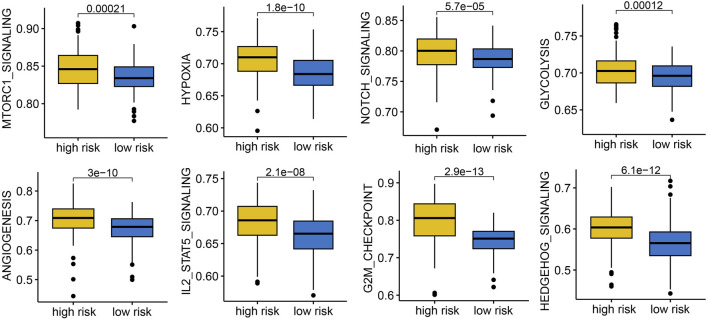
The correlation between IES-based risk score and cancer related hallmarks in STAD. STAD patients with high IES-based risk score had a higher gene set score correlated with mTORC1 signaling, hypoxia, NOTCH signaling, glycolysis, angiogenesis, IL2-STAT5 signaling, G2M_checkpoint, and hedgehog signaling.

To gain mechanistic insights, we performed functional enrichment analyses. High-risk patients showed enhanced activation of hallmark cancer pathways, including mTORC1 signaling, hypoxia, NOTCH signaling, glycolysis, angiogenesis, IL2-STAT5 signaling, G2M_checkpoint, and hedgehog signaling, indicating a more aggressive tumor phenotype (Figure 6).

### 3.8 Validation of KLF16 expression and function in STAD

**FIGURE 7:**
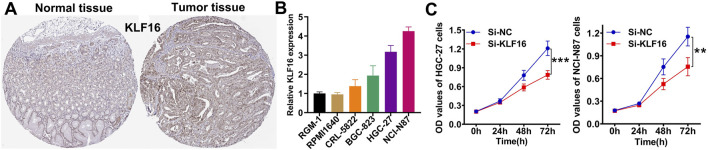
Verification of the expression and function of KLF16. **(A)** Representative immunohistochemical images of KLF16. **(B)** the mRNA level of KLF16 in normal and STAD cell lines. **(C)** Downregulation of KLF16 inhibited the proliferation of STAD cell lines.

Among the key genes comprising the IES, KLF16 was selected for further validation. Immunohistochemistry confirmed overexpression of KLF16 in STAD tissues (Figure 7A), and RT-qPCR showed elevated mRNA levels of KLF16 in most of STAD cell lines compared to normal gastric epithelial cells (Figure 7B). Knockdown of KLF16 significantly inhibited the proliferation of STAD cells, supporting its oncogenic role (Figure 7C).

## 4 Discussion

In this study, we developed a novel IES using an integrative machine learning framework, capable of accurately predicting prognosis and immunotherapy response in STAD. The model, based on immune evasion-related genes, demonstrated strong prognostic value across multiple cohorts and outperformed traditional clinicopathological features.

Many prognostic signatures have been developed in STAD. Chang et al. developed a mitochondrial-related gene signature for predicting survival in STAD (Chang et al., 2023). Necroptosis-related gene signature could predict the prognosis of STAD patients (Wang and Liu, 2021). Moreover, glutamine metabolism genes signature acted as a prognostic biomarker for STAD (Li H. et al., 2023). A 13-gene metabolic signature was associated with clinical and immune features in STAD (Ye et al., 2021). Zeng et al. also developed TGF-β signaling-related signature (Zeng et al., 2022) and anoikis- and epithelial‒mesenchymal transition-related signature (Zeng et al., 2025) for evaluating the prognosis and immunotherapy benefits in STAD. In our study, we developed a IES using an integrative machine learning framework, which have a higher C-index and AUC value compared with these gene signatures, suggesting the better performance of IES in predicting the prognosis of STAD patients.

The immune evasion phenotype has increasingly been recognized as a critical barrier to effective cancer immunotherapy (Haynes et al., 2024). Our results revealed that a low IES score is associated with enhanced anti-tumor immunity, including increased CD8^+^ T cell and dendritic cell infiltration, elevated expression of HLA genes, and upregulated immune checkpoints. CD8^+^ T cells were one of most important immune cells for anti-cancer (Chen et al., 2024). Dendritic cells are a diverse group of specialized antigen-presenting cells with key roles in the initiation and regulation of innate and adaptive immune responses (Wculek et al., 2020). These findings suggest that patients with low IES scores possess a more inflamed tumor microenvironment and may derive greater benefit from immune checkpoint blockade therapies.

In contrast, high IES scores were linked to an immunosuppressive milieu characterized by M2 macrophage dominance and diminished immune activity. Higher M2 macrophage were correlated with immunosuppressive microenvironment and poor clinical outcome (Chen et al., 2023). This immunological landscape likely contributes to resistance to immunotherapy and poor survival outcomes. Notably, our model also effectively stratified patient responses in three independent immunotherapy-treated cohorts, further supporting its clinical applicability. However, the generalizability of the IES to other cancer types in our results should be considered exploratory and that cross-tumor validation is needed. And the absence of STAD-specific immunotherapy cohort or evidence is one of the limitations of this study.

In addition to immunological insights, our analysis revealed that high IES scores correlate with the activation of oncogenic pathways such as mTOR, glycolysis, and EMT, which are known to drive tumor progression and immune resistance. Angiogenesis is a critical driver of tumor progression and metastasis in STAD (Xu et al., 2022). STAD cells undergo metabolic reprogramming, shifting towards glycolysis to enhance their survival and metastatic potential, which suggests that targeting glycolysis could be a promising therapeutic strategy for STAD (Zheng et al., 2023). These associations provide a biological rationale for the observed poor prognosis in high-risk patients and suggest potential combinatorial therapeutic targets.

We further validated KLF16 as a representative gene from the IES. Its overexpression in STAD and functional role in promoting cell proliferation indicate it may serve as both a prognostic marker and a therapeutic target. KLF16 promotes tumor growth and MYC signature in prostate cancer (Zhang et al., 2020). Moreover, KLF16 favors the tumorigenesis and progression of breast cancer by activating MAGT1 (Li et al., 2022). KLF16 promotes the proliferation and migration in bladder cancer by regulating TGFBR3 expression. In our study, silencing KLF16 significantly reduced STAD cell growth, underscoring its role in tumor biology.

## 5 Limitations

Several limitations should be acknowledged in this study. First, we excluded STAD patients who died within 3 months of diagnosis, which may have introduced a degree of selection bias. Additionally, the lack of validation in an independent clinical or prospective cohort limits the generalizability of our findings. Furthermore, the LASSO-based construction of the IES may impose constraints on model complexity, potentially affecting the accuracy and robustness of the final predictive performance. We excluded all cases with OS follow-up<90 days, which may substantially distort survival distributions and model calibration. The role of KLF16 in the immune-evasion biology was not clarified in this study, which was the direction of our subsequent research.

## 6 Conclusion

Taken together, our findings underscore the importance of immune evasion in shaping clinical outcomes in STAD. This IES not only facilitates patient risk stratification but also holds promise for guiding immunotherapy decisions and identifying patients who may benefit from alternative or combination therapies.

## Data Availability

The original contributions presented in the study are included in the article/Supplementary Material, further inquiries can be directed to the corresponding authors.
