# Supplementary material for: Machine learning based immune evasion signature for predicting the prognosis and immunotherapy benefit in stomach adenocarcinoma
Source: Front Cell Dev Biol. 2025 Sep 25;13:1656367. doi: 10.3389/fcell.2025.1656367 (PMC12507746; doi:10.3389/fcell.2025.1656367)
Supplement: Supplementary file 1 [file Table1.docx]

Supplementary Table 1. Aging and obesity-related genes using in the study

| Aging-related genes | Obesity-related genes |
| --- | --- |
| GHR  GHRH  SHC1  POU1F1  PROP1  TP53  TERC  TERT  ATM  PLAU  ERCC2  ERCC8  WRN  LMNA  IGF1R  TXN  KL  E2F1  PTPN11  NFKB2  STAT5B  STAT3  STAT5A  NRG1  HDAC3  GH1  IL7R  IGF1  IGF2  INS  NGF  IRS1  PTPN1  IRS2  AKT1  PIK3CB  NGFR  HRAS  MYC  EGFR  ERBB2  INSR  NCOR1  NBN  JUND  IL2  PDGFB  EGF  IL2RG  FOS  PDGFRB  EPOR  SST  PRKCD  PPARA  RET  PLCG2  PEX5  TCF3  PARP1  BRCA1  PIN1  PTEN  CREBBP  HIF1A  UBB  RPA1  BLM  BCL2  S100B  VCP  POLG  IGFBP3  HSP90AA1  NR3C1  EGR1  VEGFA  ABL1  BRCA2  TOP2A  TOP2B  NFKB1  TOP1  RAD51  UBE2I  TNF  PDPK1  CEBPA  CEBPB  MXI1  TGFB1  ERCC6  STK11  EP300  APTX  PML  GSK3B  HTT  PRKCA  SSTR3  HELLS  APOC3  EEF2  ERCC3  TERF1  PRKDC  CAT  ERCC5  AR  GTF2H2  XRCC5  PCNA  FEN1  FAS  TERF2  XRCC6  POLD1  BAX  RB1  EMD  GRB2  FOXO3  FOXO1  HSF1  XPA  MSRA  RECQL4  SOD2  SOD1  FOXM1  COQ7  CACNA1A  LRP2  AIFM1  UCHL1  APP  APOE  A2M  SNCG  PRDX1  PON1  RELA  IL6  RGN  ATP5O  RAD52  TOP3B  ERCC1  SIRT1  HDAC1  HSPA9  GPX1  GSR  GSS  GSTA4  GSTP1  MT-CO1  HSPD1  HSPA1A  HSPA1B  PCMT1  MAPK8  YWHAZ  PTK2B  PTK2  IL7  MAPK14  FGFR1  SP1  FLT1  JUN  MED1  MAPK9  MAPK3  HMGB1  CCNA2  HMGB2  MAP3K5  TAF1  LMNB1  SDHC  FOXO4  HESX1  PIK3R1  BSCL2  AGPAT2  BMI1  EEF1A1  TFAP2A  BDNF  CREB1  ATF2  TBP  APEX1  HBP1  BUB1B  PTGS2  HSPA8  SIN3A  CDK1  TFDP1  DDIT3  POLA1  MAPT  CTGF  HDAC2  MAX  MXD1  MDM2  SUMO1  H2AFX  HOXB7  HOXC4  JAK2  ESR1  LEP  LEPR  NFKBIA  CLU  MTOR  GHRHR  CTNNB1  PSEN1  DLL3  CDKN2A  PPP1CA  DBN1  NOG  ELN  ATR  UCP3  ZMPSTE24  TP63  UCP2  POLB  GCLC  GCLM  SIRT6  BUB3  RAE1  PMCH  MLH1  CSNK1E  STUB1  PPM1D  CHEK2  PCK1  ARHGAP1  CDC42  ARNTL  CLOCK  HIC1  PAPPA  ADCY5  PPARGC1A  GPX4  UCP1  FGF23  EFEMP1  ERCC4  CETP  PPARG  AGTR1  CISD2  EEF1E1  EPS8  KCNA3  SIRT7  SLC13A1  SOCS2  TPP2  TP53BP1  SIRT3  NCOR2  SUN1  BAK1  IGFBP2  PYCR1  TP73  CNR1  NFE2L2  CDKN1A  PDGFRA  PIK3CA  C1QA  CDKN2B  EIF5A2  MIF  DGAT1  MT1E  FGF21  HTRA2  GSK3A  NUDT1  IKBKB  SQSTM1  CDK7  GRN  SERPINE1  SPRTN  RICTOR  CTF1  TRAP1  TRPV1  NFE2L1  IFNB1  GDF11  GAGE2D  PDE6A  GABRR1  DCAF8L1  DPYSL4  SLC6A3  MMP3  RIBC2  KCNH2  HTRA3  PDX1  ATHL1  PRTG  SHC4  C21orf29  SMIM32  C1orf133  ALOX5AP  ATP6V1C1  BGN  C1QB  CAPG  CCL15  CD53  CFL1  CKB  COL1A1  CRYAB  CTSA  CTSB  CTSD  CTSK  CTSS  CTSV  CTSZ  DUSP1 | \| ALOX5AP \| \| --- \| \| ATP6V1C1 \| \| BASP1 \| \| BGN \| \| C1QB \| \| CAPG \| \| CCL15 \| \| CD53 \| \| CD68 \| \| CFL1 \| \| CKB \| \| COL1A1 \| \| CRYAB \| \| CSF1R \| \| CSTB \| \| CTSA \| \| CTSB \| \| CTSD \| \| CTSK \| \| CTSS \| \| CTSV \| \| CTSZ \| \| DAD1 \| \| DUSP1 \| \| DYNLT1 \| \| FBLN2 \| \| FCER1G \| \| FCGR2A \| \| FLNA \| \| FXYD5 \| \| GRN \| \| HCLS1 \| \| IFI27 \| \| LAPTM5 \| \| LBP \| \| LEP \| \| LGALS3 \| \| LGMN \| \| LRP1 \| \| MFGE8 \| \| MIF \| \| MRC1 \| \| MT2A \| \| NEDD8 \| \| PEA15 \| \| PFN1 \| \| PIK3R2 \| \| PITPNA \| \| PLD3 \| \| PLIN2 \| \| PLTP \| \| POSTN \| \| PSAP \| \| SEC13 \| \| SELPLG \| \| SERPINF1 \| \| TLN1 \| \| TPD52 \| \| TPM4 \| \| UCP2 \| \| AIP \| \| ARL6 \| \| ARMC5 \| \| ATRX \| \| BBS1 \| \| BLM \| \| BRAF \| \| CCDC28B \| \| CDH23 \| \| CUL4B \| \| DYRK1B \| \| GNAS \| \| HERC2 \| \| LIPE \| \| MAGEL2 \| \| MKRN3 \| \| NKAP \| \| NPAP1 \| \| NR3C1 \| \| PDE11A \| \| PRKAR1A \| \| PWAR1 \| \| PWRN1 \| \| RNPC3 \| \| SIM1 \| \| SNORD115-1 \| \| SNORD116-1 \| \| TP53 \| \| USP48 \| \| USP8 \| \| ZPR1 \| \| ADIPOQ \| \| HSD11B1 \| \| LPL \| \| PPARG \| \| RETN \| \| RXRA \| \| TNF \| \| ADNP \| \| AKT2 \| \| ALMS1 \| \| BBIP1 \| \| BBS10 \| \| BBS12 \| \| BBS2 \| \| BBS4 \| \| BBS5 \| \| BBS7 \| \| BBS9 \| \| BPTF \| \| BRD4 \| \| CEP19 \| \| CEP290 \| \| CFAP410 \| \| CFAP418 \| \| CREBBP \| \| DLK1 \| \| EP300 \| \| FMR1 \| \| GHR \| \| GJA5 \| \| GJA8 \| \| HDAC8 \| \| IFT172 \| \| IFT27 \| \| IFT74 \| \| IGF1 \| \| IGF1R \| \| IGFALS \| \| INPP5E \| \| LAS1L \| \| LZTFL1 \| \| MAN1B1 \| \| MC4R \| \| MEG3 \| \| MKKS \| \| MKS1 \| \| NIPBL \| \| NPHP1 \| \| PCNT \| \| PCSK1 \| \| PHF6 \| \| PHLDB1 \| \| PIGA \| \| POMC \| \| PSMD12 \| \| RAD21 \| \| RTL1 \| \| SCAPER \| \| SCLT1 \| \| SDCCAG8 \| \| SLC10A7 \| \| SLC7A7 \| \| SMC1A \| \| SMC3 \| \| THOC2 \| \| TRAPPC9 \| \| TRIM32 \| \| TTC8 \| \| VPS13B \| \| WAC \| \| WDPCP \| \| XRCC4 \| \| XYLT1 \| \| ZBTB20 \| \| CNNM2 \| \| ARID5B \| \| FTO \| \| IRX3 \| \| IRX5 \| \| PPARGC1A \| \| PRDM16 \| \| TBX1 \| \| UCP1 \| \| ACLY \| \| ADRB3 \| \| AGT \| \| ALDH2 \| \| ALDOA \| \| APOE \| \| ASNS \| \| ATP5ME \| \| B2M \| \| BCAT2 \| \| CDKN2C \| \| CFB \| \| CFD \| \| COX8A \| \| CYC1 \| \| CYP2E1 \| \| DBI \| \| DDT \| \| ECHS1 \| \| EEF1A1 \| \| FABP4 \| \| FDFT1 \| \| FMO1 \| \| GBE1 \| \| GNAI1 \| \| GNG11 \| \| GPD2 \| \| HBB \| \| HP \| \| LDHB \| \| MCCC1 \| \| MYLK \| \| NNAT \| \| PC \| \| PCK1 \| \| PPA1 \| \| PYGB \| \| RASD1 \| \| RBMS2 \| \| RBP4 \| \| SCD \| \| SDHB \| \| SOD1 \| \| SREBF1 \| \| THRSP \| \| TSHR \| \| UBB \| \| UCK1 \| \| UQCRC2 \| \| GAGE2D \| \| PDE6A \| \| GABRR1 \| \| DCAF8L1 \| \| DPYSL4 \| \| SLC6A3 \| \| MMP3 \| \| RIBC2 \| \| KCNH2 \| \| HTRA3 \| \| PDX1 \| \| ATHL1 \| \| PRTG \| \| SHC4 \| \| C21orf29 \| \| SMIM32 \| \| C1orf133 \| \| SHC1 \| \| TERT \| \| ATM \| \| PLAU \| \| ERCC2 \| \| ERCC8 \| \| WRN \| \| LMNA \| \| TXN \| \| KL \| \| E2F1 \| \| PTPN11 \| \| NFKB2 \| \| STAT3 \| \| STAT5A \| \| HDAC3 \| \| IL7R \| \| IRS1 \| \| PTPN1 \| |
