# Supplementary figures and images for: Machine learning based immune evasion signature for predicting the prognosis and immunotherapy benefit in stomach adenocarcinoma
Source: Front Cell Dev Biol. 2025 Sep 25;13:1656367. doi: 10.3389/fcell.2025.1656367 (PMC12507746; doi:10.3389/fcell.2025.1656367)

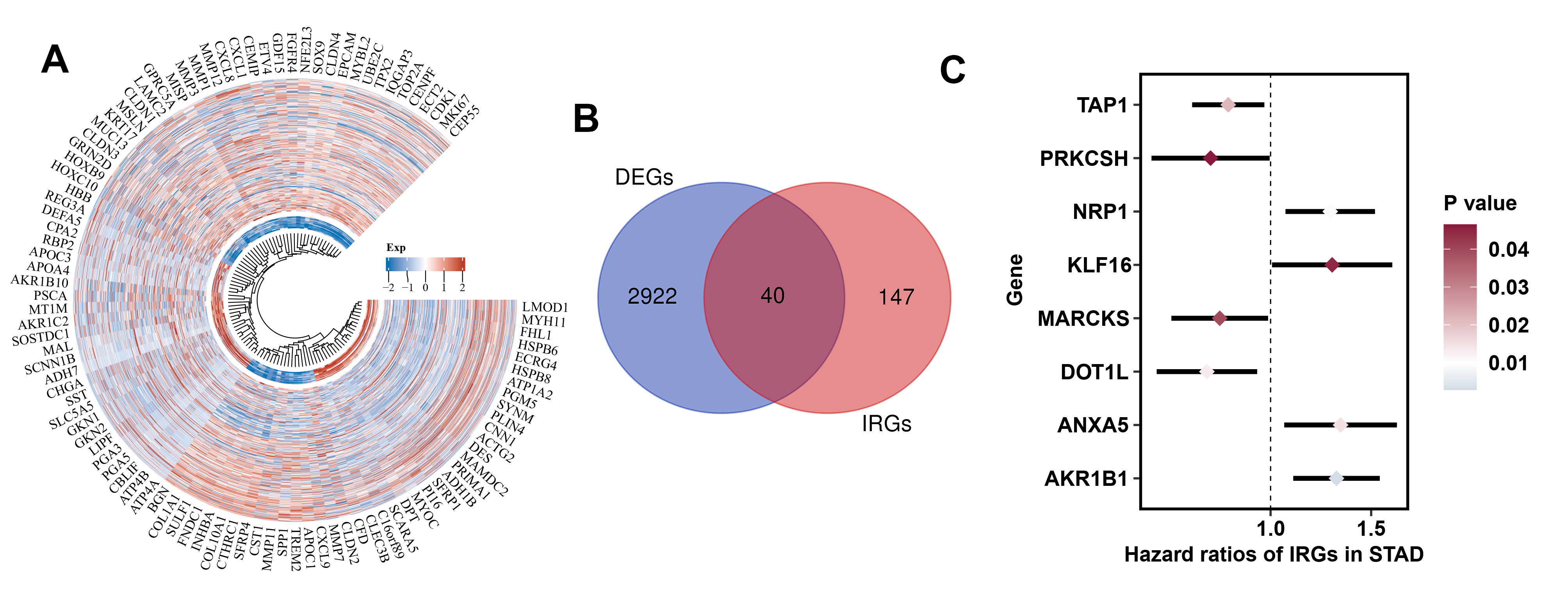

Supplement: Supplementary file 2 [file Image1.jpeg]
